# Polymorphism of **μ**-Opioid Receptor Gene (OPRM1:c.118A>G) Might Not Protect against or Enhance Morphine-Induced Nausea or Vomiting

**DOI:** 10.1155/2013/259306

**Published:** 2013-02-04

**Authors:** Li-Kuei Chen, Shiou-Sheng Chen, Chi-Hsiang Huang, Hong-Jyh Yang, Chen-Jung Lin, Kuo-Liong Chien, Shou-Zen Fan

**Affiliations:** ^1^Department of Anesthesiology, National Taiwan University Hospital, National Taiwan University College of Medicine, Taipei 10048, Taiwan; ^2^Department of Anesthesiology, National Taiwan University Hospital Hsin-Chu Branch, Hsinchu City 30059, Taiwan; ^3^Department of Urology, School of Medicine, National Yang-Ming University, Taipei 112, Taiwan; ^4^Department of Surgery, Taipei City Hospital, Renai Branch, Taipei 103, Taiwan; ^5^Department of Internal Medicine, National Taiwan University Hospital Hsin-Chu Branch, Hsinchu City 30059, Taiwan; ^6^College of Public Health, National Taiwan University, Taipei 10020, Taiwan

## Abstract

A cohort, double blind, and randomized study was conducted to investigate the effect of a single nucleotide polymorphism of the **μ**-opioid receptor at nucleotide position 118 (OPRM1:c.118A>G) on the association with the most common side effects (nausea or vomiting) induced by intravenous patient control analgesia (IVPCA) with morphine, including incidence and severity analysis. A total of 129 Taiwanese women undergoing gynecology surgery received IVPCA with pure morphine for postoperative pain relief. Blood samples were collected and sequenced with high resolution melting analysis to detect three different genotypes of OPRM1 (AA, AG, and GG). All candidates 24 h postoperatively will be interviewed to record the clinical phenotype with subjective complaints and objective observations. The genotyping after laboratory analysis showed that 56 women (43.4%) were AA, 57 (44.2%) were AG, and 16 (12.4%) were GG. The distribution of genotype did not violate Hardy-Weinberg equilibrium test. There was no significant difference neither between the severity and incidence of IVPCA morphine-induced side effects and genotype nor between the association between morphine consumption versus genotype. However, there was significant difference of the relation between morphine consumption and the severity and incidence of IVPCA morphine-induced nausea and vomiting. The genetic analysis for the severity and incidence of IVPCA morphine-induced nausea or vomiting showed no association between phenotype and genotype. It might imply that OPRM1:c.118A>G does not protect against IVPCA morphine-induced nausea or vomiting.

## 1. Introduction

IVPCA with pure morphine at present clinical practice was still widely used for postoperative pain management by providing excellent analgesic effect [1, 2]; however, the high incidence of some annoying side effects, especially nausea or vomiting induced by IVPCA morphine, may limit its clinical implication. The most common side effects of IVPCA morphine are nausea or vomiting, with some other less common like pruritus (2% to 10%) [3], urinary retention and respiration depression [4, 5]. The incidence of postoperative nausea or vomiting has been reported from 20% to 30% and the incidence of severe nausea and vomiting around 0.1% [6–8]. The prophylactic protocol or treatment regimen for opioid-induced nausea or vomiting had been elucidated and studied recently with many publications [9–19]. Previous studies investigating the association between IVPCA morphine and the genetic variability of human *μ*-opioid receptor gene had focused on the difference of morphine consumption or analgesic effects [20–26]. Nevertheless, the association between the side effects induced by IVPCA morphine (especially nausea or vomiting) and the genetic variability of human *μ*-opioid receptor gene had never been elucidated thoroughly and specifically. We designed and conducted this cohort, double blind, randomized study to investigate the effect of OPRM1:c.118A>G on the nausea or vomiting induced by IVPCA morphine following total abdominal hysterectomy analgesia, including incidence and severity analysis.

## 2. Materials and Methods

### 2.1. Patient Profile and Anesthetic Procedure

This study was approved by the Ethic Institute Review Board of the National Taiwan University Hospital after obtaining a written informed consent. This was a population-based, prospective observational study, with the double blind designed in data analysis. A total of 129 American Society of Anesthesiologists physical status I or II Taiwanese women who underwent total abdominal hysterectomy (TAH) and received IVPCA morphine for postoperative pain control were recruited into this study in the 12-month period from August 1, 2007 to July 31, 2008. All the recruited women could understand and describe the pain score. Those women were excluded for any of the following reasons: contraindications for IVPCA morphine, complaint of nausea or vomiting before the operation, a history of significant cardiovascular disease, renal disease, diabetes, hepatic disease, or chronic pain with taking pain medication. A standardized, general anesthesia technique was used for all patients. For induction of anesthesia, 2 *μ*g/kg fentanyl, 2 mg/kg propofol, and 0.15 mg/kg cisatracurium were used. For maintenance of anesthesia during operation, cisatracurium and inhaled anesthetic desflurane at a low flow rate of 0.5 L/min were applied. Residual neuromuscular block was antagonized with 2.5 mg neostigmine and 1.0 mg atropine, and patients were extubated at the end of surgery. After extubation, patients were transferred to the postanesthesia care unit for observation at least 1 hour and then transferred back to ward. All of the recruited women, attending anesthesiologists for practicing anesthesia and analgesia, and the well-trained investigator were blinded to the genotype at time of surgery because genotyping was determined only later in the laboratory.

### 2.2. Postoperative Pain Management, Assessment, and Data Collection

At the postanesthesia care unit, patients were asked every 15 min whether pain medication indicated to reduce their 10 cm VAS pain score <3, until they became alert enough to use the IVPCA pump. The morphine solution provided in the PCA pump contained 250 mL normal saline and 100 mg pure morphine. The pump was set to deliver a 1 mg bolus of morphine solution with a lockout time of 5 min and a maximum dose of 15 mg within a 4 h limit without a background. Overdose was prevented by limiting the total dose administrated within a given period of time. The total amount of consumed morphine for 72 h after the operation was recorded by the PCA device and was recorded using an Abbott TRW printer model TP 40 (Abbott Life Care Infuser; Chicago, IL, USA). PCA was started immediately after patients were alert to control the PCA machine in the postanesthesia care unit and was stopped 72 h after the operation. A trained investigator would interview the patient one time per day during the first 72 h postoperatively. If patients still felt wound pain (10 cm VAS pain score over 3) even after we gave the loading dose as 3 mg of morphine and doubled the bolus dose as 2 mg, those cases would be excluded. This investigator would also explain those possible side effects induced by IVPCA morphine to patients and record those side effects (especially focus on nausea and vomiting) as mild or severe. For those patients with severe side effects, this investigator would conduct effective management immediately following anesthesiologist's order.

Data related to patients' age, weight, height, history of previous operation, ASA class, and wound pain score were collected. Itching is defined as the sensation that provokes desire to scratch the skin over the whole body. Nausea is defined as the sensation of having an urge to vomit and vomiting was defined by the number of episodes of retching with or without expulsion of fluids from the stomach. Urinary retention is defined as patients could not void by themselves and urinal catheterization is indicated. Dizziness is defined as having a whirling sensation and a tendency to fall and lethargy is defined as a state of sluggishness, inactivity, and apathy. All of the above side effects (including nausea, vomiting, itching, dizziness) were scored on a severity scale as the following: severe = the number of episodes > 3 and medical treatment is indicated, mild = the number of episodes ≦ 3 and medical treatment is not indicated, and none as the number of episodes = 0. The incidence of the above side effects is defined as the number of episodes taking place during the observation period. Patients who had severe nausea or vomiting or dizziness were treated with prochlorperazine 5 mg; patients with severe itching were treated with diphenhydramine HCL 4 mg.

### 2.3. Laboratory Analysis

**Figure 1:**
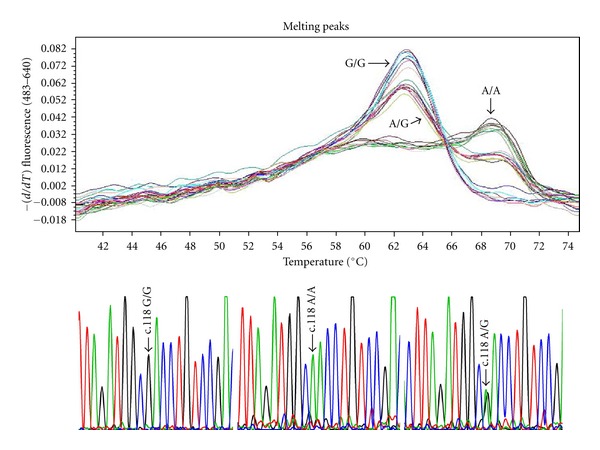
The figure from high resolution melting analysis to detect three different genotypes (AA, AG, and GG) by using the LightCycler 480 Gene Scanning Software.

Genomic DNA was extracted from 3 mL of peripheral blood cell samples with a Puregene DNA Isolation Kit (Gentra Systems, Minneapolis, MN, USA), according to the manufacturer's instructions. Real-time PCR and data analysis were performed in a LightCycler 480 Real-Time PCR System and with LightCycler 480 software, using LightCycler FastStart DNA Master HybProbe Kit. The reactions in a final volume of 10 *μ*L consisted of 0.25 *μ*M concentrations of each primer (forward, 5′-GTAGAGGGCCATGATCGTGAT-3′; and reverse, 5′-GCTTGGAACCCGAAAAGTCT-3′), 0.25 *μ*M concentrations of each probe (forward, 5′-CCCGGTTCCTGGGTCAACTTGTCC-3′; and reverse, 5′-CTTAGATGGCAACCTGTCCGACC-3′) 1 *μ*L of Fast Start DNA master hybridization probe reaction mixture (Roche), 5 mM MgCl_2_, and 1 *μ*L of genomic DNA. Pipet 10 *μ*L PCR products into each well of the LightCycler 480 (Roche Applied Science) Multiwell Plate. Load the multiwall Plate in LightCycler 480 instrument and start the melting program. High resolution melting was performed at 95°C for 1 min, 40°C for 1 min, 6.5°C for 1 s, and acquisitions at 95°C. The data was evaluated using the LightCycler 480 Gene Scanning Software (Figure 1). The blood samples were sequenced for genotypes: wild-type A118 homozygous (AA), mutant heterozygous (AG), and mutant G118 homozygous (GG), which were first treated as individual variables.

### 2.4. Statistical Analysis

The nonnormal distributed variable, morphine consumptions were summarized by median and interquartile range (IQR). Morphine consumptions in various genotypes or side effects with two categories were compared by the nonparametric Mann-Whiney test; morphine consumptions in various genotypes or side effects with three categories were compared by the nonparametric Kruskal-Wallis test with Bonferroni correction for post-hoc comparisons. The categorical variables were summarized by count and percentages, and the associations between them were tested by Fisher's exact test. The genetic frequency was analyzed with using 10,000 permutations to approximate and exact *P* value for the HWE (Hardy-Weinberg equilibrium) test, as well as 1,000 bootstrap samples to obtain the confidence interval for the allele frequencies and one-locus Hardy-Weinberg disequilibrium (HWD) coefficients. The goodness of fit in Hardy-Weinberg equilibrium was tested with chi-square test. Statistical significance was set at 0.05. Statistical analyses were performed with the SPSS 15.0 software package (SPSS Inc., Chicago, IL, USA).

## 3. Results

A total of 132 women were enrolled in this study, and due to incomplete analgesia (VAS > 3), the 3 were excluded remaining 129 women were analyzed in this study. The genotyping after laboratory analysis showed that 56 women (43.4%) were AA, 57 (44.2%) were AG, and 16 (12.4%) were GG. This sample size has a power greater than 80% to detect a 10% difference in those side effects among three different genotype groups. The allelic frequencies for the A and G alleles were 65.5% and 34.5%, respectively. The distribution of genotypes and allelic frequencies did not violate Hardy-Weinberg equilibrium (Table 5, *P* = 0.969).

### 3.1. Side Effects versus Genotype

The association between the severity of IVPCA-morphine-induced side effects and three genotypes was shown in Table 1, with all the *P* value > 0.5. The association between the incidences of IVPCA-morphine-induced side effects and three genotypes was shown in Table 2, with all the *P* value > 0.5 In the analysis of the severity and incidence, no significant association was found between IVPCA morphine-induced side effects and three genotypes (Tables 1 and 2).

### 3.2. Morphine Consumption versus Genotype

The genetic analysis using autosomal dominant, autosomal recessive, and codominant model of inheritance to evaluate the differences between genotypes and morphine consumptions was shown in Table 3, without significant difference found between IVPCA morphine consumption and genotypes.

### 3.3. Morphine Consumption versus Side Effects

The relation between morphine consumption and the side effects was summarized in Table 4. Patients with the side effect of nausea had significantly less morphine consumptions than those without occurring nausea (21.0 mg (16.0, 33.1) versus 29.0 mg (21.0, 39.0), *P* = 0.010). A similar result was observed in the side effect of vomiting: patients with the side effect of vomiting had significantly less morphine consumptions than those without occurring vomiting (19.1 mg (15.0, 29.0) versus 29.5 mg (20.0, 37.1), *P* = 0.004). In the comparisons for morphine consumption between various degrees of vomiting, patients with severe vomiting had significantly less morphine consumptions than those without occurring vomiting (19.1 mg (11.1, 23.1) versus 29.5 mg (20.0, 37.1)).

## 4. Discussion

Previous pharmacogenetic studies for morphine have been focused on the investigation of morphine consumption variety between the different genotypes [20, 23, 25, 27]. However, the specific genetic study for those side effects induced by morphine was limited, except Romberg et al. group reporting the association between respiratory depression induced by opioid with OPRM1:c.118A>G [21]. Nausea or vomiting, the most common side effect induced by intravenous morphine for postoperative analgesia was observed with different phenotypes in incidence and severity among different patients. Previous studies have supplementary mentioned about the association between OPRM1:c.118A>G with nausea or vomiting induced by morphine, without definite or with defect conclusion [20, 23, 25, 27]. 

The possible mechanisms of opiates inducing nausea or vomiting might be attributed as follows: direct activation of the chemoreceptor trigger zone (CTZ) in the area postrema of the medulla, with the action conveyed to the vomiting center; increased sensitivity of vestibular function and indirect stimulation of the CTZ, with action conveyed to the vomiting center; decreased stomach motility; prolongation of gastric emptying time; and increased possibility of esophageal reflux. However, the incidence and severity of nausea or vomiting induced by intravenous morphine had been reported differently according to previous papers [7, 8]. The differences might be attributed to the route of administrating the opioids [28]; the gender factor (the incidence higher in female patients [28]), the different ethnics [11, 29], and so on. Especially, the study by Hirayama et al. clearly indicated that postoperative nausea and vomiting (PONV) with morphine therapy developed in more than 60% of Japanese patients after surgery [11]. We may reasonably hypothesize that OPRM1:c.118A>G could have played a role in protecting against or increasing the severity and incidence of nausea or vomiting induced by IVPCA morphine.

Reviewing previous published papers, we found there might be too many confounding factors affecting the diversified incidence and severity of PONV. Patient-related factors might be attributed to the following: age, gender, obese patients, history of motion sickness and/or previous postoperative nausea, anxiety, gastroparesis, and different operative procedures. Anesthetic-related factors associated with emesis might be attributed to those confounding factors: preanesthetic medication, gastric distension and suctions, different anesthetic techniques (general anesthesia, regional anesthesia, or monitored anesthesia care), and postoperative factors (including painful sensation, dizziness feeling, ambulation, oral intake and PCA device with opioids). Most previous studies had been focused on evaluating the relationship between morphine consumption and genetic variations by using IVPCA morphine for different operations, with the subsidiary conclusion that there was no association between side effects and genotype polymorphism [20, 23, 25, 27]. At present, there is not yet a complete and well-designed genetic association study to particularly evaluate those most common side effects induced by epidural morphine. However, pharmacogenetic studies for nausea or vomiting specifically induced by IVPCA morphine should exclude those confounding factors, with definite confirmation that nausea or vomiting should be induced by morphine only. The results from previous pharmacogenetic studies [20, 23, 25, 27] for nausea or vomiting induced by morphine have the following defects due to not excluding those confounding factors. Most of studies [20, 23, 25, 27] recorded their data for side effects since patients were sent to postanesthesia care unit and started to use IVPCA morphine. Therefore, it could be impossible to get the reliable and true data for the incidence and severity of nausea or vomiting induced by IVPCA morphine, without excluding the confounding factors (such as preanesthetic medication, gastric distension and suctions, and different anesthetic techniques). In one study [23], they analyzed the genetic association between the IVPCA morphine consumption after intrathecal morphine with OPRM1:c.118A>G. They concluded that OPRM1:c.118A>G had a significant effect on pain perception, analgesic requirement, and nausea/vomiting for patient who received PCA intravenous morphine after intrathecal morphine. However, apparently there were some confounding factors with inadequate study design in patient selection to influence the genetic variation analysis for the severity and incidence of those side effects induced by morphine, since those patients in the study received intrathecal 0.1 mg morphine during spinal anesthesia first, then followed by IVPCA morphine for postoperative pain management. The underlying mechanisms for the incidence and severity of those side effects induced by neuroaxial or parenteral morphine were different, and those side effects did not have distinct proportional relationship with opioid dosage neither [30–33]. For example, the most common and severe side effect induced by intrathecal morphine could be pruritus, and the most common and severe side effect induced by intravenous morphine could be nausea or vomiting. It might be inferred importantly and reasonably that the study design and patient selection of the genetic variation evaluation for those side effects induced by morphine should be homogenous, unitary such as the single intervention design by only parenteral or neuroaxial morphine for postoperative analgesia alone. 

In our study design for recording those side effects induced by IVPCA morphine and patient selection, we have tried our best to exclude those confounding factors that would have the high possibility to affect the results we collected for the analysis. We recruited more homogenous criteria with Taiwanese female who received the same operation (TAH) with the same general anesthesia protocol, and used IVPCA morphine only for postoperative analgesia. In the recruiting criteria, we excluded those patients with any systemic disease or medication associated with nausea or vomiting, with the history of PONV after surgery and complaints of nausea or vomiting before surgery. We started to record those side effects (including the severity and incidence analysis) induced by IVPCA morphine one time per day 24 hours after surgery to exclude those confounding factors (such as preanesthetic medication, gastric distension and suctions and different anesthetic techniques). Furthermore, we excluded those patients with inadequate analgesia (VAS > 3), medications related to PONV, dizziness feeling, or ambulation influence to exclude those postoperative confounding factors. At the meantime, the same well-trained nursing observer who was blind to the genetic analysis data visited all the patients one time per day and recorded all the data. Under this selection criteria and study design, we might reasonably confirm that those side effects were definitely and purely induced by IVPCA morphine. Compared with previous similar pharmacogenetic studies [20, 23, 25, 27] for morphine with lower incidence (around 15%) and less severe of nausea or vomiting, the overall incidence of nausea (81/129, 62.8%), vomiting (58/129, 45%), and nausea or vomiting (82/129, 63.6%) induced by IVPCA in our study was much more compatible with and similar to previous published papers [11, 34–39] and our previous experience. During the data analysis, accidently we found that patients with the episode of nausea and/or vomiting had significantly less morphine consumptions (Table 4) and patients with severe nausea or vomiting had significantly less morphine consumptions (Table 4), although there was no significant difference between IVPCA morphine consumption and genotypes (Table 3). However, we still need more clinical information to detect if there was any relationship among the three dimensions: total morphine consumption, different genotypes, and incidence and severity of nausea or vomiting induced by IVPCA morphine.

In summary, our study might be the most integrated and thorough investigation on the genetic relationship between OPRM1:c.118A>G and nausea or vomiting induced by morphine. The major finding of our study is that not only the incidence of nausea or vomiting induced by IVPCA morphine following TAH analgesia but also the severity was not associated with A118G polymorphism of OPRM1:c.118A>G in Taiwanese women with definite and clear conclusion. It might imply that OPRM1:c.118A>G does not protect against nor enhance nausea or vomiting induced by IVPCA morphine in severity and incidence.

## Figures and Tables

**Table 1:** The association between the severity of IVPCA morphine-induced side effects and genotype.

		Total (*n* = 129)	Genotype			*P* value
			A/A (*n* = 56)	A/G (*n* = 57)	G/G (*n* = 16)	
Nausea	Severe	36 (27.9%)	15 (26.8%)	16 (28.1%)	5 (31.3%)	0.987
	Mild	45 (34.9%)	19 (33.9%)	21 (36.8%)	5 (31.3%)	
	None	48 (37.2%)	22 (39.3%)	20 (35.1%)	6 (37.5%)	
Vomiting	Severe	28 (21.7%)	11 (19.6%)	14 (24.6%)	3 (18.8%)	0.910
	Mild	30 (23.3%)	13 (23.2%)	12 (21.1%)	5 (31.3%)	
	None	71 (55.0%)	32 (57.1%)	31 (54.4%)	8 (50.0%)	
Itching	Severe	3 (2.3%)	2 (3.6%)	1 (1.8%)	0 (0.0%)	0.976
	Mild	11 (8.5%)	5 (8.9%)	5 (8.8%)	1 (6.3%)	
	None	115 (89.1%)	49 (87.5%)	51 (89.5%)	15 (93.8%)	
Dizziness	Severe	10 (7.8%)	5 (8.9%)	4 (7.0%)	1 (6.3%)	0.672
	Mild	51 (39.5%)	20 (35.7%)	22 (38.6%)	9 (56.3%)	
	None	68 (52.7%)	31 (55.4%)	31 (54.4%)	6 (37.5%)	

**Table 2:** The association between the incidences of IVPCA morphine-induced side effects and genotype.

		Total	Genotype			*P* value
			A/A (*n* = 56)	A/G (*n* = 57)	G/G (*n* = 16)	
Nausea	Yes	81 (62.8%)	34 (60.7%)	37 (64.9%)	10 (62.5%)	0.935
	No	48 (37.2%)	22 (39.3%)	20 (35.1%)	6 (37.5%)	
Vomiting	Yes	58 (45.0%)	24 (42.9%)	26 (45.6%)	8 (50.0%)	0.882
	No	71 (55.0%)	32 (57.1%)	31 (54.4%)	8 (50.0%)	
Nausea or Vomiting	Yes	82 (63.6%)	35 (62.5%)	37 (64.9%)	10 (62.5%)	0.967
	No	47 (36.4%)	21 (37.5%)	20 (35.1%)	6 (37.5%)	
Itching	Yes	14 (10.9%)	7 (12.5%)	6 (10.5%)	1 (6.3%)	0.857
	No	115 (89.1%)	49 (87.5%)	51 (89.5%)	15 (93.8%)	
Dizziness	Yes	61 (47.3%)	25 (44.6%)	26 (45.6%)	10 (62.5%)	0.469
	No	68 (52.7%)	31 (55.4%)	31 (54.4%)	6 (37.5%)	
Lethargy	Yes	12 (9.3%)	7 (12.5%)	4 (7.0%)	1 (6.3%)	0.579
	No	117 (90.7%)	49 (87.5%)	53 (93.0%)	15 (93.8%)	

**Table 3:** Tests for the relations between morphine consumption versus genotype.

		Morphine consumption (mg)	*P* value
Genotype	A/A	22.6 (14.5, 34.1)	0.430
	A/G	23.1 (19.0, 40.1)	
	G/G	26.1 (20.0, 35.6)	
Genotype	A/A	22.6 (14.5, 34.1)	0.203
	A/G or G/G	24.0 (19.0, 38.1)	
Genotype	A/A or A/G	23.0 (16.0, 36.7)	0.499
	G/G	26.1 (20.0, 35.6)	

**Table 4:** Tests for the relations between morphine consumption versus side effects.

		Morphine consumption (mg)	*P* value
Nausea	Severe	20.5 (13.6, 35.1)	0.033*
	Mild	21.0 (16.0, 32.1)	
	None	29.0 (21.0, 39.0)	
Nausea	Yes	21.0 (16.0, 33.1)	0.010*
	No	29.0 (21.0, 39.0)	
Vomiting	Severe	19.1 (11.1, 23.1)^†^	0.014*
	Mild	20.6 (15.5, 32.1)	
	None	29.5 (20.0, 37.1)	
Vomiting	Yes	19.1 (15.0, 29.0)	0.004*
	No	29.5 (20.0, 37.1)	
Nausea or vomiting	Yes	21.0 (16.0, 34.0)	0.018*
	No	28.6 (21.0, 39.2)	

*Indicated that there is significant difference in morphine consumption between various categories in the corresponding side effect.

^†^Indicated that there is significant difference in morphine consumption between those with severe vomiting and without vomiting.

**Table 5:** Test for Hardy-Weinberg equilibrium.

	The observed frequency	The expected frequency in a state of Hardy-Weinberg equilibrium	*P* value
Genotype			
A/A	56 (43.4%)	55.4 (42.9%)	0.969
A/G	57 (44.2%)	58.3 (45.2%)	
G/G	16 (12.4%)	15.4 (11.9%)	
